# Redo Gastric Bypass following internal herniation with gangrenous roux limb, in second trimester pregnancy: How safe?

**DOI:** 10.1016/j.ijscr.2022.107728

**Published:** 2022-10-11

**Authors:** Siddharth Sankar Das, Zaid AbdulAziz Ghulam, Feras Hamid Al Khitab, Farah Ibrahim B. Juma, Walid Zakaria Mohd Bandok

**Affiliations:** Department of Surgery, Dubai Hospital, Dubai, United Arab Emirates

**Keywords:** Gastric bypass, Internal hernia, Pregnancy, Peterson's defect, Intestinal ischemia

## Abstract

**Introduction and importance:**

Internal herniation following Gastric bypass is a serious life-threatening complication, needs prompt diagnosis and intervention. Internal herniation in later part of pregnancy can endanger life of both mother and fetus if not managed diligently.

**Case presentation:**

30-year young lady with post gastric bypass status with 26 weeks of pregnancy presented with intestinal obstruction. Clinically she was suspected to have internal herniation. She was carrying a viable healthy intrauterine baby. Emergency laparotomy performed and the gangrenous roux limb was resected and Re-do gastric bypass was created. She delivered a healthy female baby at 37+ weeks.

**Clinical discussion:**

Internal hernias after RYGB are more common in pregnant women due to cephalad displacement of intestines and creation of potential hernial spaces due to excess fat loss. Pregnancy with post RYGB status with intestinal obstruction, possibilities of internal hernia need to be excluded. In case non-viable intestinal loops, reconstruction of bypass possible. Post operatively cares with nutritional supplements play major role for fetal growth in advanced stage of pregnancy.

**Conclusion:**

Internal hernia during pregnancy needs prompt intervention which can save of life mother as well as intrauterine baby.

## Highlights

• Internal hernia following gastric bypass is a serious life-threatening condition.
• Internal hernias after RYGB are more common in pregnant women.
• Patients with internal hernia usually presents with epigastric pain, nausea, and vomiting.
• Reconstruction of gastric bypass after resection of ischemic bowel following internal hernia

## 1 Introduction

Roux-en-Y Gastric bypass (RYGB) has been accepted as one of the standard bariatric surgery procedures for morbid obesity. Worldwide obese female patients prefer more to undergo bariatric surgery even at younger age to attend normal body mass index. After the procedure, with weight loss majority women prefer marriage and subsequent childbirth. In becomes challenging to manage nutritional demand of pregnant mother and the intrauterine baby during pregnancy. Also handling any complication related to bariatric surgery during pregnancy always carries risk of life to baby as well as mother. Internal herniation is a serious complication can be life threatening for mother and baby during pregnancy. It needs prompt diagnosis and immediate intervention to minimize the risk. This article, we want to describe our experience of maternal and fetal outcome from pregnancy with reconstruction of RYGB after resection of gangrenous elementary limb due to internal hernia after primary RYGB.

This work has been reported in line with SCARE Criteria [1].

## 2 Presentation of case

**Fig. 1:**
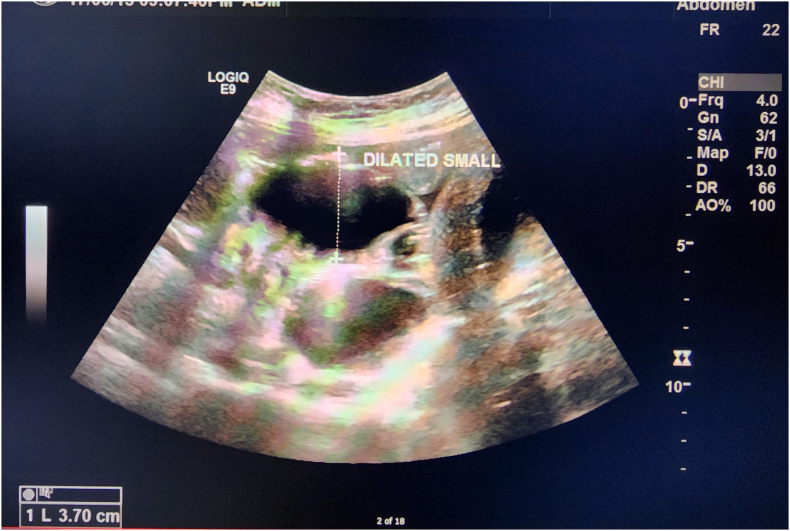
Ultrasound abdomen-showing dilated small bowel loops with free fluid in abdomen.

**Fig. 2:**
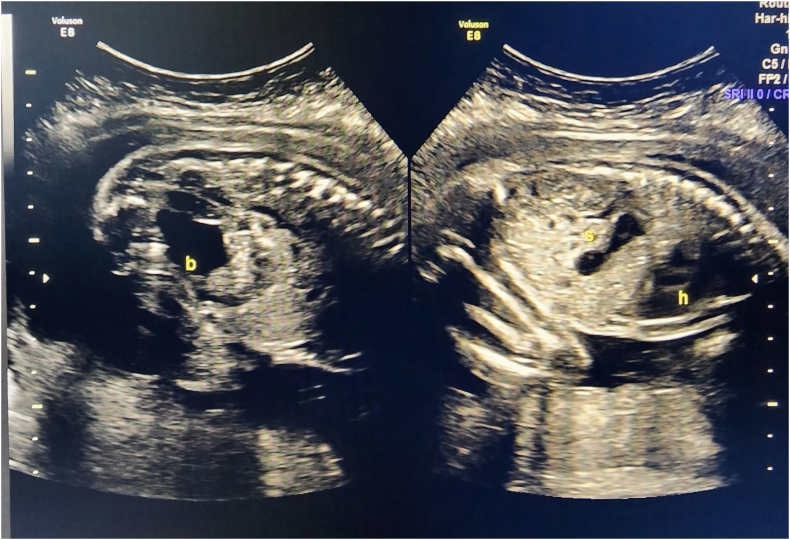
Fetal scan -Obstetrics growth scan showing fetal growth and well-being.

A 30-year young lady with 26 weeks of pregnancy presented to emergency with complain of pain upper abdomen for 3 days, vomiting for 2 days followed by hematemesis for one day. She had undergone Roux-en-Y gastric bypass four years back in Saudi Arabia. She was initially admitted in another hospital and due to lack of surgical division and neonatal ICU was shifted to our hospital, for management of baby in case of premature delivery. On investigations showing raised WBC count, lactic acidosis, and CRP level. Ultrasound abdomen was done which was showing multiple dilated small bowel loops (Fig. 1) displaced to upper part of abdomen by gravid uterus with intra-abdominal free fluid. She was also bearing a 26 week healthy intrauterine baby. Ultrasound done to know fetal wellbeing, showing healthy intrauterine baby (Fig. 2). Gynecologist assessed the mother and baby and were satisfied with baby growth but were concerned about mother's condition. CT scan abdomen was not done due to denied consent for procedure by mother to prevent radiation hazard to fetus. On insertion of Nasogastric tube foul smelling hemorrhagic fluid aspirated giving suspicion to have internal herniation and gangrenous bowel loop. Emergency surgery planned with gynecologist, pediatrician, and neonatal Intensivist on board. Emergency diagnostic laparoscopy performed which was showing dilated gangrenous roux limb with internal herniation through Peterson's defect. The internal hernia was tried to reduce laparoscopically but failed due to reduced intra-abdominal space due to space occupying gravid uterus. Immediately converted to midline laparotomy. The gangrenous roux limb from Gastro-jejunal(G-j) junction to Jejuno-jejunal(J-j) anastomosis was resected out. Re do gastric bypass was performed creating new roux limb and forming re gastrojejunostomy. Jejuno-jejunostomy performed 80 cm from Duodeno-jejunal (DJ) flexure. Re-do gastric bypass was performed as per the personal request of patient, while obtaining consent before surgery. She was very much insisting to maintain her gastric bypass status. Post operatively patient was on total parenteral nutrition support and recovered smoothly. The baby was monitored regularly during admission by gynecologist showing healthy viability. She was discharged on 10th post-operative day. Post Re-do Bypass patient and her fetus were monitored and followed regularly by dietician, gynecologist, and bariatric team. She delivered a healthy term female baby of 3.172 kg at 37+ weeks.

**Fig. 3:**
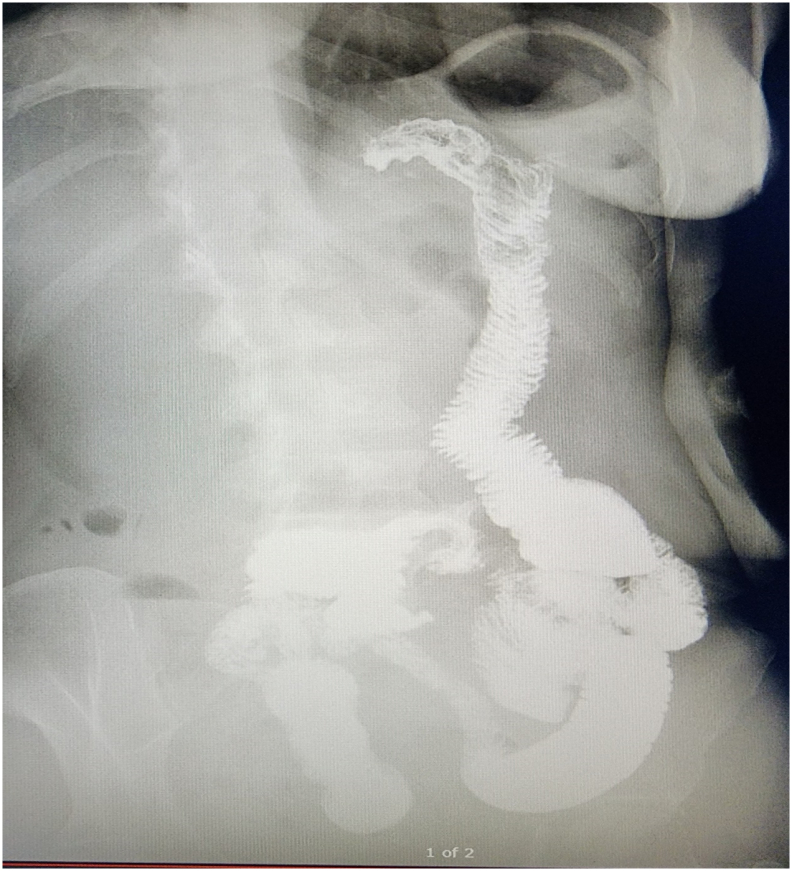
Oral Gastrograffin study during follow up, 1 year after redo bypass, giving impression about gastric pouch size and flow of contrast through Roux limb to distal loops.

Patient reduced from 142 kg, Body mass Index (BMI)-58.43 kg/m^2^, pre bypass status to 60.5 kg, BMI-24.89 kg/m^2^, pre pregnancy in four years post-surgery. During pregnancy she gained weight to reach 66 kg, BMI-27.16 kg/m^2^, while presented to us with internal hernia with intestinal ischemia and underwent resection of intestine and redo bypass. Redo Bypass constructed as patient has requested preoperatively to preserve her gastric bypass status. Post re-do Bypass she delivered healthy female child of weight 3.172 kg at 37 weeks. She exclusively breast fed her baby for 6 months. She achieved weight of 51.4 kg, BMI-21.1 kg/m^2^, at two years follow up [Fig. 3]. She is happy, to deliver a healthy baby, while retaining her gastric bypass status despite of the tragedy during her later part of pregnancy.

## 3 Discussion

Bariatric surgery is a popular way among morbidly obese women to achieve a healthy BMI, which subsequently improves chance of fertility and better pregnancy outcome [2], [12], [13]. Two of the most popular bariatric surgery are sleeve gastrectomy and gastric bypass, both having promising results.

Patients undergoing gastric bypass are more likely to have short term and long-term complications in comparison to sleeve gastrectomy [3]. Common complications after RYGB are like anemia, nutritional deficiencies, anastomosis stricture, internal hernia, dumping syndrome etc. [4]. Internal hernias after RYGB are more common in pregnant women due to cephalad displacement of bowel loops due to pelvic and abdominal space occupancy by gravid uterus along with creation of potential hernial spaces due to excess fat loss after surgery [4], [5]. Laparoscopic gastric bypass in most centers done as antecolic fashion which creates Peterson's defect (created space between transverse mesocolon and antecolic elementary limb)and jejuno-jejunostomy mesenteric defect. In our case the herniation was through Peterson's defect with twisting of elementary limb mesentery with compromised vascularity resulting gangrene of roux limb requiring resection of gangrenous segment.

Most of the pregnant patients with internal hernia usually presents with epigastric pain, nausea, and vomiting [9]. Any pregnant lady with post RYGB status presenting with upper abdominal pain should alert the surgeons about possibilities of internal hernia, which needs to be excluded first without delay. In our case the patient had symptoms for three days and getting treatment in an obstetrics specialty hospital without involvement of surgical team, hence delayed diagnosis resulted gangrenous bowel.

Post Gastric bypass, with reduction of weight, intrabdominal visceral fat density reduces. Even if during RYGB, potential internal hernia defects closed, not enough to prevent internal herniation subsequently, as the gap between sutures widen up. During pregnancy, the gravid uterus displaces the intestines cranially and increases intra-abdominal pressure [2], [9], [10], [11]. This increases the chance of internal hernia most describes in second and third trimester of pregnancy [6], [15], [16], [17].

Computer tomography (CT) with contrast is the investigation of choice for detection of internal hernia. Ct scan shows some classical sign of mesenteric swirl sign and superior mesenteric vein compression suggestive of internal hernia [7], [14]. Due to potential risk of radiation hazard, the risk and benefit of CT scan needs to be explained to patient and consent needs to be taken. Our patient was reluctant to give consent for CT scan with contrast and on insertion of NG tube foul smelling hemorrhagic fluid aspirated, indicating possible gangrenous bowel loop and decision of emergency surgery planned without CT scan abdomen.

After resection of gangrenous elementary limb along with J-J anastomosis, we reconstructed new gastrojejunostomy and Jejunojejunostomy restoring bypass status as patient was keen to maintain her bypass status. Even with bowel resection and reconstruction of RYGB in second trimester of pregnancy, post operatively with regular follow up with nutritionist, patient continued her pregnancy till 37+ weeks and delivered a healthy baby. The extra protein requirement during pregnancy along with bypass surgery needs intensive oral liquid protein supplement along with other micronutrients. With the new anastomosis performed with ongoing pregnancy, the nutritional supplements play major role to sustain the fetal growth in advanced stage of pregnancy. The patient education and motivation also play important role to encourage intake not only for her health, but also for her fetus. Patient should be frequently monitored by both dieticians to monitor adequate nutritional supplements and by gynecologist to monitor fetal growth. There is always increased risk of cesarean section [8] which patient must be explained. Our patient delivered vaginally with episiotomy. These special cases need combined monitoring and support from bariatric team, dietician, and gynecologist to deliver a healthy baby.

## 4 Conclusion

Internal hernia following gastric bypass is a serious life-threatening condition, needs urgent intervention [4]. Pregnant women with post RYGB status, presenting with abdominal pain, getting admitted to Institutions without bariatric unit or without surgical team, should take immediate surgical opinion to rule out internal hernia, to prevent any morbidity. Proactive management to diagnose and timely intervention can prevent lethal complications like intestinal ischemia. Reconstruction of gastric bypass after resection of ischemic bowel is a challenging decision but feasible with healthy outcome. These special cases need intensive post-surgery support from nutrition and gynecology department for healthy delivery of a new life.

## CRediT authorship contribution statement

**Siddharth Sankar Das**: Primary assistance, data collection, article writing, review literatures.

**Zaid AbdulAziz Ghulam**: Primary surgeon, final editing.

**Feras Hamid Al Khitab**: data collection, article writing, review literatures.

**Farah Ibrahim B Juma**: data collection, article writing, review literatures.

**Walid Zakaria Mohd Bandok**: data collection, article writing, review literatures.

## Funding

This research did not receive any specific grant from funding agencies in the public, commercial, or not-for-profit sectors.

## Ethical approval

The ethical committee approval was not required given the article type (case report).

## Consent

Written informed consent was obtained from the patient for publication of this case report and accompanying images. A copy of written consent is available for review by the editor-in-chief of this journal on request.

## Registration of research studies

Not applicable.

## Guarantor

Siddharth Sankar Das.

## Provenance and peer review

Not commissioned, externally peer-reviewed.

## Declaration of competing interest

The authors state that they have no conflicts of interest for this report.
